# Seizure Like Activity During Permanent Pacemaker Implantation: A Case of Suspected Venous Air Embolism With Acute Hypoxia

**DOI:** 10.1002/ccr3.73689

**Published:** 2026-10-06

**Authors:** Sanjida Ansari, Murari Dhungana

**Affiliations:** ^1^ Shahid Gangalal National Heart Center Kathmandu Nepal

**Keywords:** acute hypoxia, permanent pacemaker implantation, seizure‐like activity, venous air embolism

## Abstract

Venous air embolism is a rare complication of pacemaker implantation. A 78 year old man developed sudden generalized stiffness, seizure‐like activity, and severe hypoxia (oxygen saturation 70%) during lead manipulation without hypotension or arrhythmia. Prompt recognition, high‐flow oxygen, and temporary procedural interruption led to complete recovery without neurological sequelae.

**Key Clinical Message**

- Venous air embolism during permanent pacemaker implantation may present atypically with sudden hypoxia and transient seizure‐like activity without hemodynamic instability.
- Prompt recognition during lead manipulation and immediate administration of high‐flow oxygen are crucial.
- Any unexplained intra‐procedural desaturation should raise suspicion to prevent potentially serious complications and ensure favorable outcomes.

## 1 Introduction

Venous air embolism (VAE) is a recognized but relatively uncommon complication encountered during invasive medical and surgical procedures. It occurs when air enters the venous circulation and subsequently travels to the right heart and pulmonary vasculature potentially leading to significant cardiopulmonary compromise. Although VAE has been widely described in neurosurgical, obstetric, and central venous catheter‐related settings, its occurrence during cardiac implantable electronic device (CIED) procedures including permanent pacemaker implantation remains under‐reported and often under‐recognized [1, 2].

Permanent pacemaker implantation is a routinely performed procedure for the management of bradyarrhythmias such as complete heart block. Despite its overall safety profile, procedural complications may occur including pneumothorax, lead dislodgement, infection, vascular injury, and rarely air embolism [2]. The mechanism of VAE in this setting typically involves inadvertent entrainment of air through venous access sites, introducer sheaths, or during lead manipulation. Factors such as negative intrathoracic pressure during spontaneous inspiration, hypovolemia, and improper handling of venous access systems can increase the risk of air entry into the circulation [3].

Once air enters the venous system, it is transported to the pulmonary circulation where it can obstruct pulmonary blood flow, increase pulmonary artery pressures, and create ventilation‐perfusion mismatch. The clinical manifestations of VAE depend on the volume and rate of air entry as well as patient specific factors. Classical presentations include sudden onset dyspnea, chest pain, cough, hypotension, and in severe cases, cardiovascular collapse [3, 4]. However, smaller volumes of air may produce subtle or transient symptoms making diagnosis challenging in the procedural setting.

An important but less commonly recognized aspect of VAE is its potential to produce neurological manifestations. These may arise secondary to cerebral hypoxia due to impaired pulmonary gas exchange or less frequently from paradoxical embolization in the presence of intracardiac shunts such as a patent foramen ovale. Neurological symptoms can range from confusion and agitation to seizure‐like activity, which may mimic primary neurological disorders or other procedural complications such as local anesthetic systemic toxicity [4]. This atypical presentation can delay diagnosis and appropriate management, particularly in electrophysiology laboratories where patients are often under conscious sedation.

The true incidence of clinically significant VAE during CIED implantation is not well established partly due to under‐detection and lack of systematic reporting. Large registry‐based studies focusing on device‐related complications suggest that while major complications are relatively infrequent, rare events such as air embolism may be overlooked unless they produce overt clinical consequences [2]. Consequently, heightened clinical awareness and vigilance are essential, especially during critical procedural steps such as venous access and lead advancement.

Early recognition of VAE is crucial as prompt management can significantly reduce morbidity and prevent progression to life‐threatening complications. Immediate administration of high‐concentration oxygen, cessation of further air entry, and appropriate patient positioning remain the cornerstone of treatment. Preventive measures, including careful handling of introducer systems, minimizing open hub time, and maintaining adequate venous pressure, are equally important in reducing the risk of this complication [3].

In this report, we describe a case of suspected venous air embolism occurring during permanent pacemaker implantation presenting atypically with seizure‐like activity and acute hypoxia in the absence of hemodynamic instability. This case underscores the importance of recognizing non‐classical presentations of VAE and reinforces the need for prompt clinical suspicion during device implantation procedures.

## 2 Case Presentation

A 78‐year‐old male with a known diagnosis of complete heart block (Figure 1) and Chronic Obstructive Pulmonary Disease (Figure 2) was admitted for elective permanent pacemaker implantation. At baseline, the patient was hemodynamically stable with an oxygen saturation of 98% on room air. There was no history of prior neurological disorders and preprocedural evaluation was unremarkable.

**FIGURE 1:**
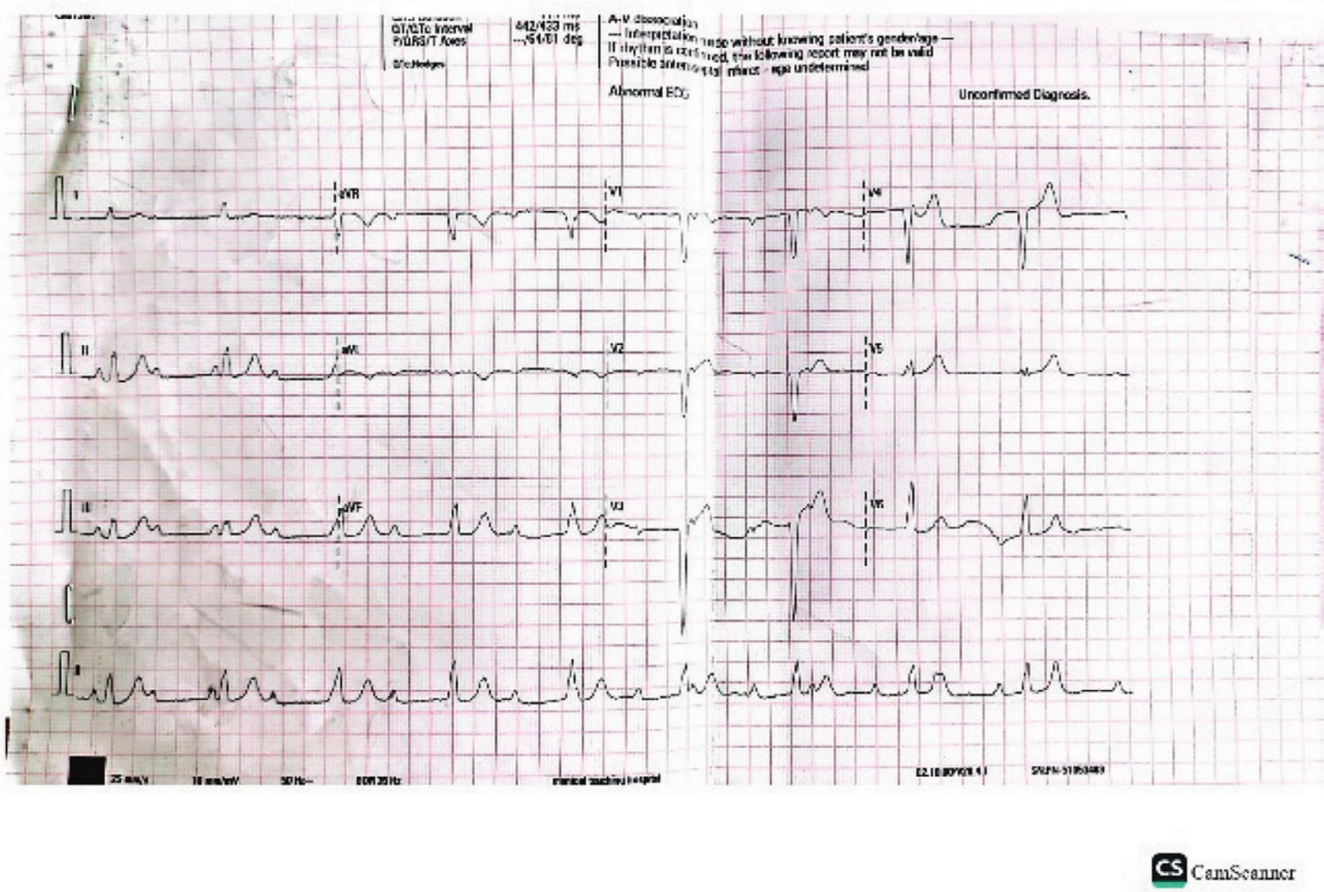
Complete heart block.

**FIGURE 2:**
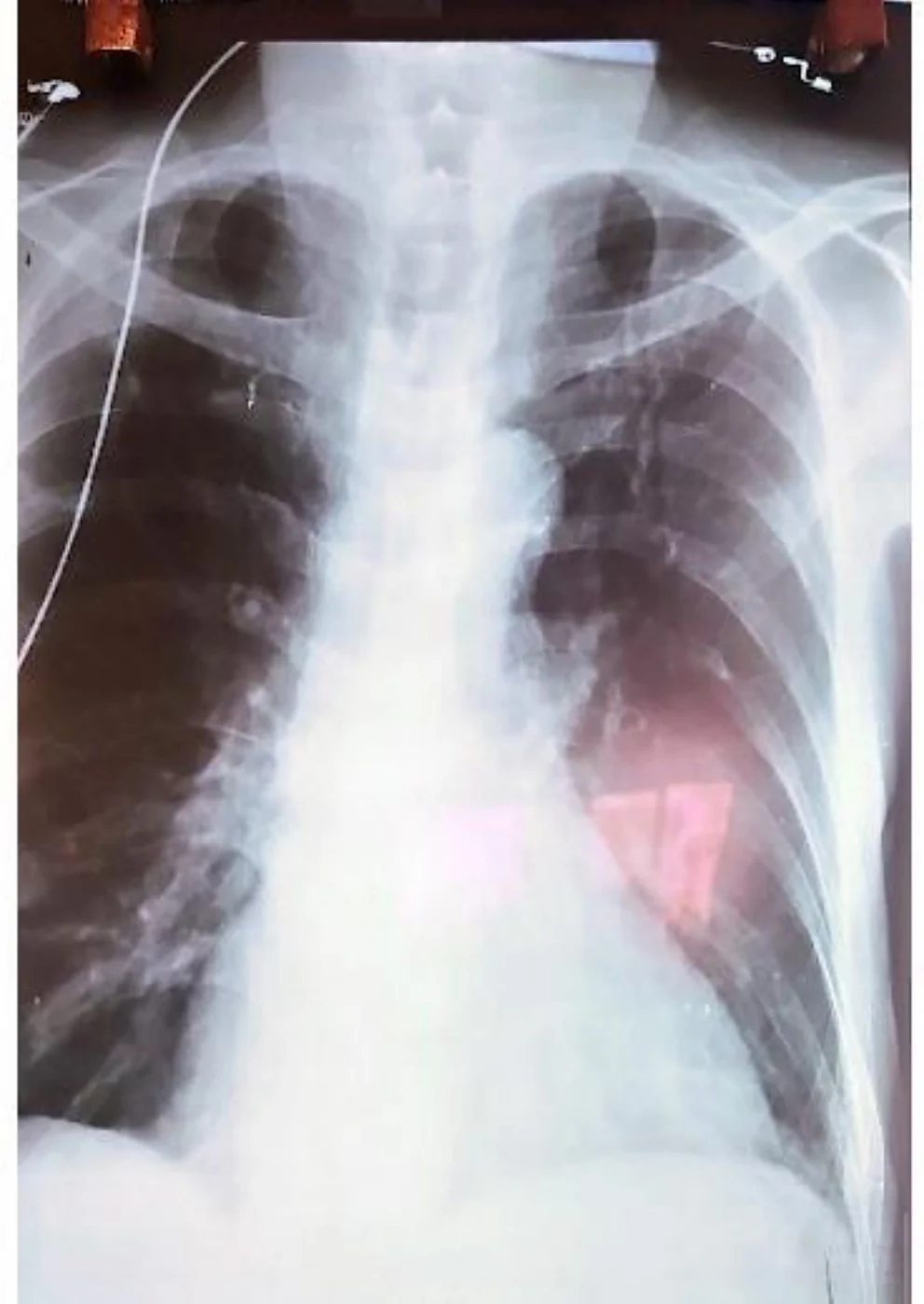
Chest X‐ray before Implantation.

The procedure was performed under local anesthesia with continuous electrocardiographic and pulse oximetry monitoring. Left axillary venous access was successfully obtained and initial steps of lead placement were uneventful. However, during advancement of the pacing lead, the patient acutely developed generalized body stiffness accompanied by involuntary, seizure‐like movements. This was immediately followed by a sudden drop in oxygen saturation to 70%. Notably, there was no associated hypotension or arrhythmia on continuous monitoring.

Given the abrupt onset of hypoxia temporally related to lead manipulation, venous air embolism was suspected clinically. The procedure was promptly halted and high‐flow oxygen (100%) was administered. Although Trendelenburg positioning is typically recommended in such scenarios, it could not be implemented due to the presence of temporary pacing support. No additional pharmacological intervention was required. The Fluoroscopic X‐ray view was taken but did not show any presence of air in pulmonary artery (Figure 3).

**FIGURE 3:**
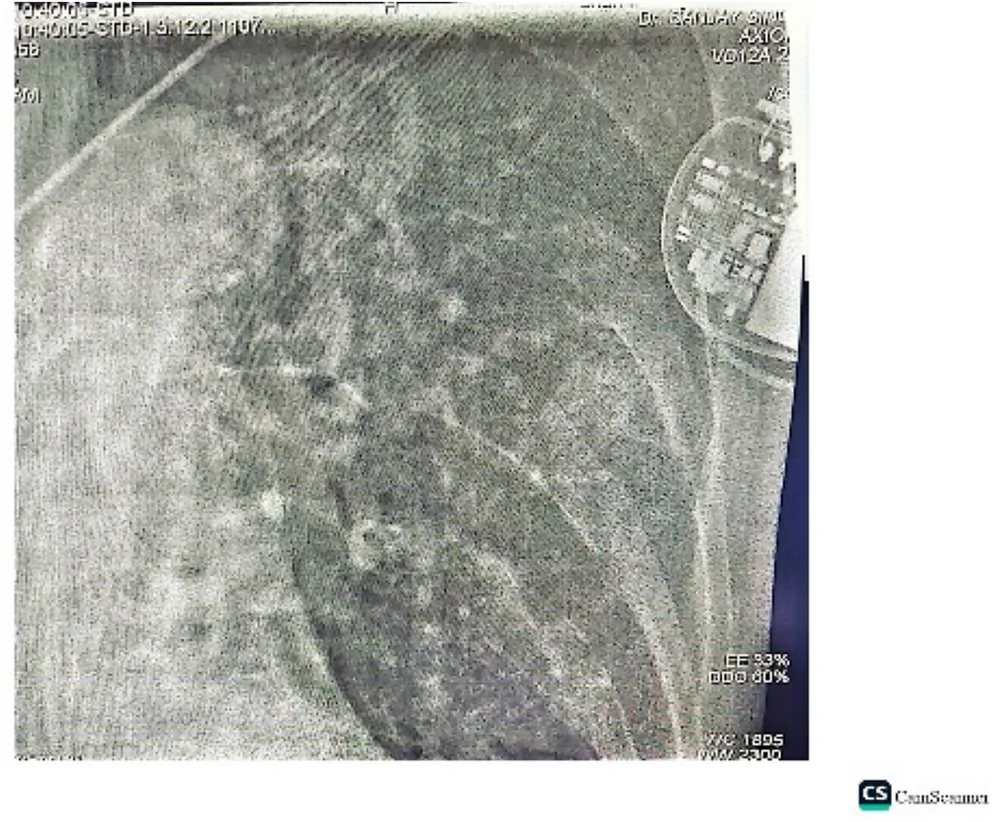
Fluoroscopic X‐ray view shows no air in pulmonary artery.

The patient demonstrated rapid clinical improvement with normalization of oxygen saturation and complete resolution of neurological manifestations within a short duration. The procedure was subsequently resumed and completed successfully without further complications (Figure 4).

**FIGURE 4:**
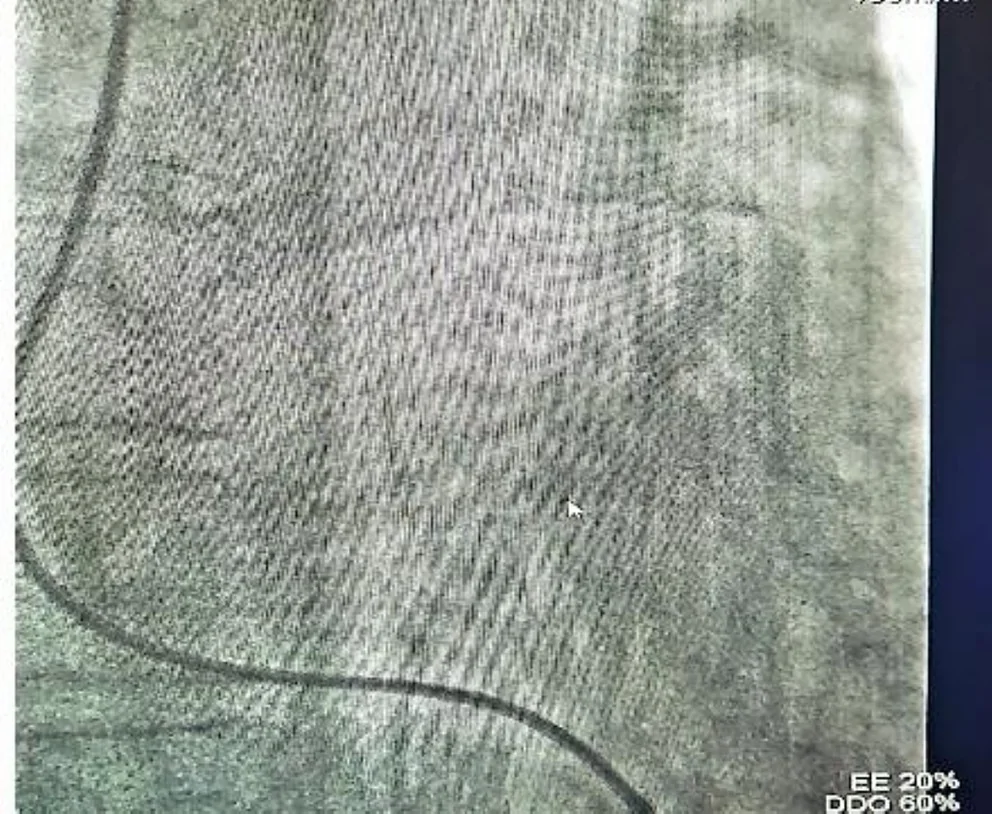
Fluoroscopic view at end of implantation.

Continuous ECG monitoring during the event did not reveal any malignant arrhythmias and pulse oximetry confirmed transient but significant desaturation. Due to the acute and self‐limiting nature of the episode, further investigations such as echocardiography or arterial blood gas analysis were not performed.

Differential diagnoses considered included venous air embolism, hypoxia‐induced seizure‐like activity, local anesthetic systemic toxicity, Acute exacerbation of Pulmonary Disease and vasovagal reaction. There was no pneumothorax which was checked on fluoroscopy repeatedly. The temporal association with venous instrumentation, absence of cardiovascular instability and rapid resolution with oxygen therapy favored venous air embolism as the most likely diagnosis.

Post procedurally, the patient remained stable with no recurrence of symptoms. He was monitored throughout the hospital stay and discharged without any neurological deficit. Follow up evaluation did not reveal any delayed complications (Figures 5 and 6).

**FIGURE 5:**
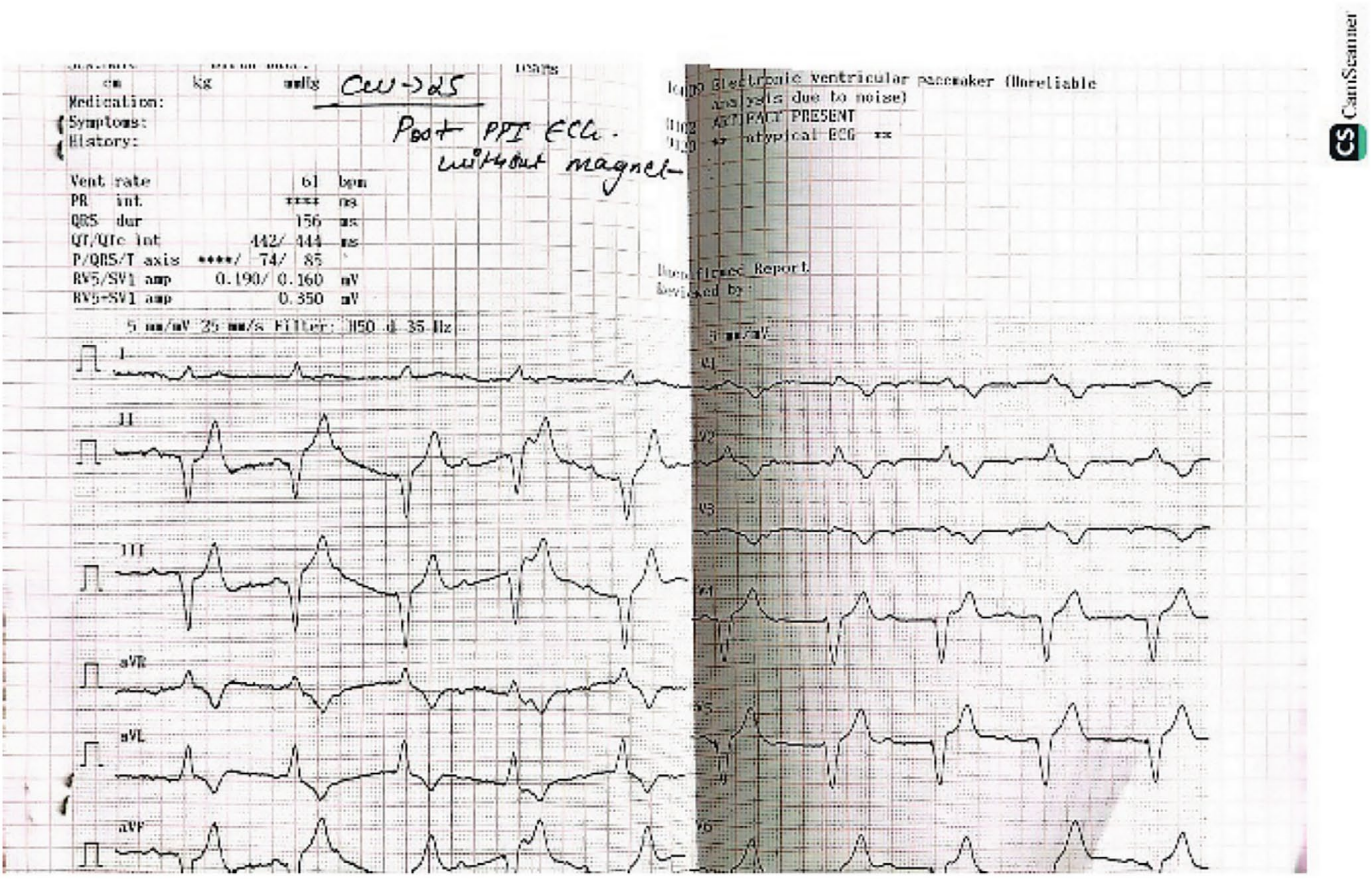
ECG after implantation.

**FIGURE 6:**
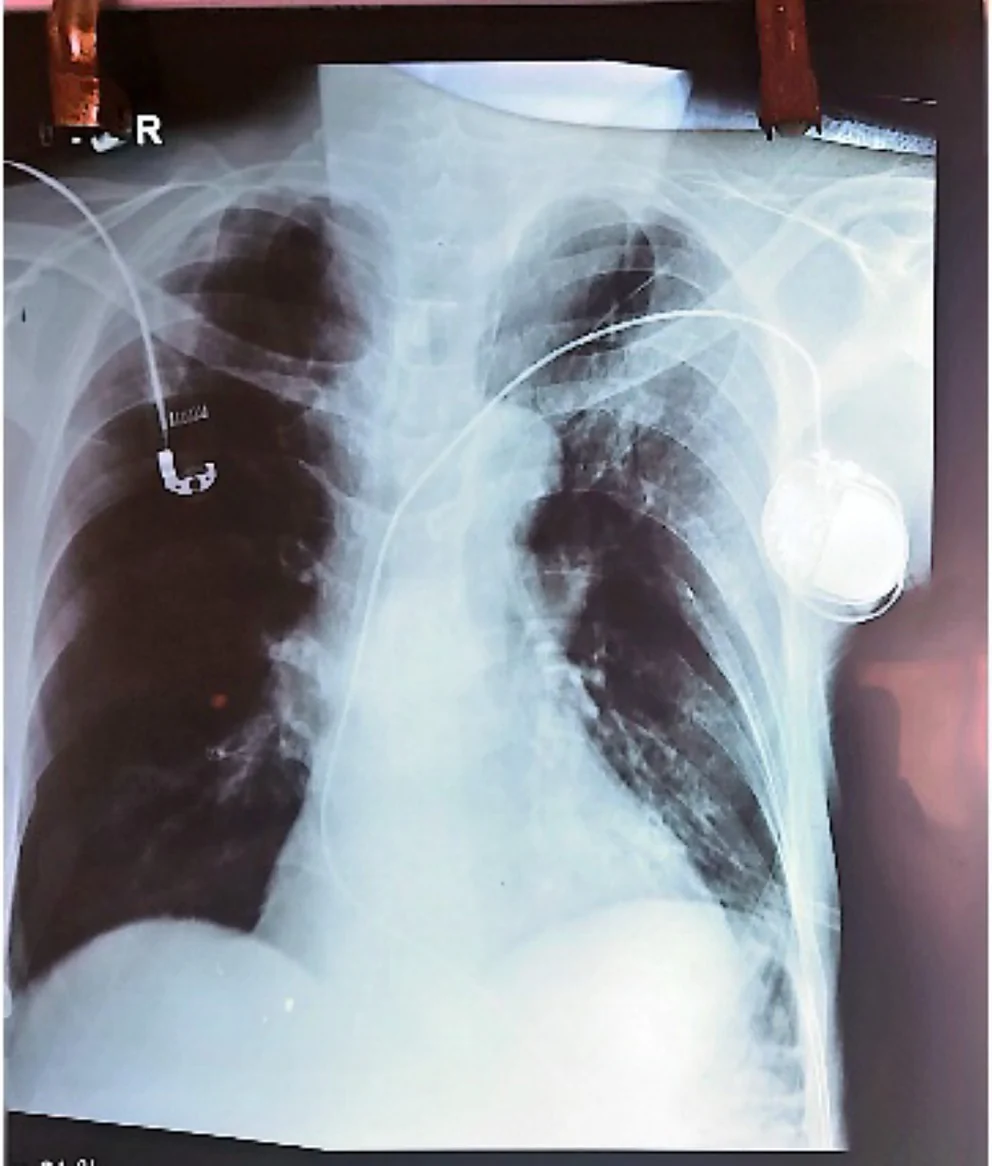
Chest X‐ray after Implantation.

## 3 Discussion

This case illustrates an atypical intra‐procedural presentation of suspected venous air embolism (VAE) during permanent pacemaker implantation, characterized by transient seizure‐like activity and acute hypoxia in the absence of hemodynamic instability. While VAE is a recognized complication of invasive procedures, its presentation with predominant neurological features during cardiac device implantation remains uncommon and may lead to diagnostic uncertainty.

A key distinguishing feature in this case is the temporal relationship between lead manipulation and the abrupt onset of symptoms. The immediate development of generalized stiffness and seizure‐like movements followed by significant oxygen desaturation strongly supports an acute intraprocedural event. In contrast to classical descriptions of VAE, which often include hypotension or cardiovascular collapse, this patient remained hemodynamically stable throughout. This highlights that even small volumes of entrained air can produce significant hypoxia sufficient to cause transient cerebral dysfunction without overt circulatory compromise [1, 3].

The absence of arrhythmia and rapid resolution of symptoms with high‐flow oxygen further supports VAE as the most likely diagnosis. Among the differentials, local anesthetic systemic toxicity typically presents with central nervous system excitation followed by cardiovascular instability, which was not observed. Similarly, vasovagal reactions are usually associated with bradycardia and hypotension rather than isolated hypoxia with neurological manifestations. Hypoxia‐induced seizure‐like activity remains a plausible mechanism; however, in this context, it is more likely secondary to pulmonary vascular obstruction caused by air embolism rather than a primary neurological event [4].

This case underscores the importance of recognizing hypoxia as an early and sometimes the only indicator of VAE during device implantation. Continuous pulse oximetry proved critical in identifying the event, as oxygen desaturation preceded full clinical appreciation of the complication. The rapid improvement following oxygen administration further reinforces the role of supportive management in suspected VAE even in the absence of confirmatory imaging or laboratory investigations.

Another important practical consideration highlighted by this case is the limitation of ideal positioning. Although Trendelenburg positioning is commonly recommended to reduce the risk of air migration, it was not feasible due to the presence of temporary pacing. This reflects real world procedural constraints and emphasizes that clinicians must rely on prompt recognition and immediate supportive measures when standard preventive or therapeutic strategies cannot be fully implemented.

From a procedural standpoint, this case reinforces the need for meticulous attention during venous access and lead manipulation. Even brief exposure of the venous system to atmospheric pressure may allow air entrainment, particularly in spontaneously breathing patients. As large registries of cardiac device implantation focus primarily on more common complications, rare events such as VAE may remain under‐reported despite their clinical relevance [2].

The clinical implication is clear; any sudden, unexplained desaturation during pacemaker implantation should be presumed to be due to air embolism until proven otherwise. Early suspicion allows for rapid intervention, which in this case resulted in complete recovery without neurological sequelae. Increased awareness among electrophysiologists and device implanters is essential to avoid misdiagnosis and delays in management.

## 4 Differential Diagnosis

The acute onset of generalized stiffness, seizure‐like activity, and abrupt oxygen desaturation during pacemaker lead manipulation necessitated consideration of several intra‐procedural complications. The leading diagnosis was venous air embolism given the temporal association with venous instrumentation and sudden hypoxia without hemodynamic collapse.

Other important differentials included hypoxia‐induced seizure‐like activity where cerebral hypoxemia secondary to acute ventilation‐perfusion mismatch could explain transient neurological manifestations. However, this is considered a secondary phenomenon rather than a primary diagnosis.

Local anesthetic systemic toxicity (LAST) was also considered, as it may present with central nervous system excitation, seizures, and cardiovascular instability following local anesthetic administration. The absence of prodromal symptoms such as perioral numbness, tinnitus, altered mental status prior to hypoxia, and the lack of cardiovascular compromise made this diagnosis less likely.

A vasovagal reaction was another consideration; however, this typically presents with bradycardia, hypotension, and diaphoresis rather than isolated hypoxia with seizure‐like activity, making it less consistent with the observed clinical course.

Given the patient's age, acute exacerbation of chronic obstructive pulmonary disease (AECOPD) was also considered in the differential diagnosis. AECOPD can present with sudden worsening of gas exchange, leading to hypoxia, agitation, and occasionally confusion or decreased consciousness. However, the prior COPD history, presence of wheezing or increased airway resistance during monitoring, and the abrupt onset immediately following venous manipulation made this a close differential diagnosis in the present case.

Less likely considerations included myocardial ischemia or arrhythmic events which can cause sudden hemodynamic instability and hypoperfusion; however, continuous ECG monitoring revealed no malignant arrhythmias or ischemic changes during the episode.

Overall, the clinical context, timing with venous access, isolated hypoxia, and rapid response to oxygen therapy strongly favored venous air embolism as the most probable diagnosis.

## 5 Unique Clinical Features

Sudden onset of generalized stiffness with seizure‐like activity occurring immediately during pacemaker lead manipulation accompanied by abrupt isolated hypoxia (SpO_2_ 70%) without hypotension or arrhythmia and rapid complete neurological recovery following high‐flow oxygen therapy.

## 6 Management Implications

This case highlights several important management implications for cardiac device implantation procedures. First, it emphasizes the need for a high index of suspicion for venous air embolism (VAE) when sudden, unexplained oxygen desaturation occurs during venous access or lead manipulation even in the absence of hypotension or arrhythmia. Early recognition is critical as clinical deterioration can be rapid but reversible if managed promptly.

Immediate administration of 100% high‐flow oxygen remains the cornerstone of treatment. Oxygen therapy not only corrects hypoxemia but also promotes nitrogen washout from intravascular air bubbles thereby reducing embolus size and improving pulmonary perfusion. Prompt cessation of the procedural step associated with air entry is equally essential to prevent further embolization.

Although patient positioning such as Trendelenburg or left lateral decubitus is traditionally recommended to limit cerebral and pulmonary air migration, this may not always be feasible in real‐world settings, particularly in patients requiring temporary pacing support. In such situations, rapid supportive management should not be delayed.

This case also underscores the importance of meticulous preventive strategies during device implantation including careful handling of venous sheaths, minimizing open hub time, and maintaining adequate intrathoracic venous pressure. Continuous pulse oximetry is essential as early desaturation may be the only warning sign.

Finally, awareness among operators is crucial to avoid misdiagnosis of seizure‐like activity as primary neurological or drug‐related events. Early suspicion and standardized response protocols can significantly reduce morbidity and ensure safe procedural outcomes.

## 7 Conclusion

Venous air embolism during permanent pacemaker implantation is a rare but important and potentially reversible complication that may present atypically with isolated hypoxia and transient seizure‐like activity, even in the absence of hemodynamic instability. This case emphasizes that sudden intra‐procedural oxygen desaturation during venous access or lead manipulation should be considered VAE until proven otherwise. Prompt recognition and immediate administration of 100% oxygen along with cessation of the offending procedural step can result in rapid clinical recovery and prevent neurological or cardiopulmonary sequelae. Sudden unexplained hypoxia with transient neurological symptoms during pacemaker implantation should raise immediate suspicion of venous air embolism, and early high‐flow oxygen therapy is critical for complete recovery.

## Author Contributions

**Sanjida Ansari:** conceptualization, investigation, writing – original draft, methodology, validation, visualization, writing – review and editing, software, formal analysis, data curation, resources, project administration. **Murari Dhungana:** conceptualization, investigation, resources, supervision, data curation, visualization, formal analysis.

## Funding

The authors have nothing to report.

## Ethics Statement

The study adhered to ethical guidelines and was conducted in accordance with the Declaration of Helsinki. Institutional ethical approval was not required for this case report.

## Consent

Written informed consent was obtained from the patient for the publication of this case report and accompanying images.

## Conflicts of Interest

The authors declare no conflicts of interest.

## Data Availability

The data supporting the findings of this study are available within the manuscript and its Supporting Information. All relevant clinical details, diagnostic images, and pathological findings have been included to ensure transparency and reproducibility.
